# Are virtual consultations suitable for patients with vulval disease? A multicentre audit of outcomes in the COVID‐19 pandemic

**DOI:** 10.1002/ski2.178

**Published:** 2022-10-10

**Authors:** Fiona M. Lewis, Sheila M. McSweeney, Jeanne Wendling, Micheline Moyal‐Barracco

**Affiliations:** ^1^ St John's Institute of Dermatology Guy's & St Thomas' Hospital London UK; ^2^ 149 bis rue Biomet Paris France; ^3^ 4 rue Léon Delhomme Paris France

## Abstract

**Background:**

During the COVID‐19 pandemic, virtual consultation (VC) was used to replace in‐person consultations. This raises specific questions when dealing with vulval conditions.

**Objectives:**

To assess the feasibility and the efficiency of VC with and without supplementary imaging, in patients with vulval conditions, and to evaluate the images provided as an aid to diagnosis.

**Methods:**

This prospective multicentre audit took place in three specialized vulval clinics in London and Paris. Anonymized data on patients' clinical characteristics, consultation characteristics (including the number and quality of any supplementary images provided) and consultation outcomes (diagnostic certainty and physician satisfaction) were collected. Characteristics and outcomes in those with or without supplementary imaging were compared amongst both new and follow‐up consultations.

**Results:**

A total of 316 VCs were included. In total, 18.7% (*n* = 59) were new patient consultations and 81.3% (*n* = 257) were follow‐up. Supplementary imaging (photographs and/or video recordings) were provided by 28.5% (*n* = 90) of the total cohort. Median photographic quality was significantly higher on a five‐point Likert‐type scale when photographs were taken by a third party as opposed to the patient themselves (4 vs. 3, Mann‐Whitney *U*‐test, *p* < 0.0001). There was no association between the provision of supplementary imaging and diagnostic certainty amongst new patient consultations. However, a higher proportion of follow‐up patients who provided supplementary imaging received definitive management decisions (*χ* ^2^ test, *p* < 0.001) and physician satisfaction with these consultations, as measured on a five‐point Likert‐type scale, was significantly higher (Mann‐Whitney *U*‐test, *p* < 0.0001). Furthermore, median physician satisfaction scores ≥4 were observed in follow‐up consultations for candidiasis, lichen simplex/eczema and vulvodynia.

**Conclusions:**

Although in‐person consultation remains the gold standard of care, VC may have a role in the management of selected patients with vulval disease. It is possible to provide good‐quality photographs for clinical assessment, particularly with the help of a third party and follow‐up patients with an established, cancer‐unrelated diagnosis may be best suited for this consultation modality.

## Graphical abstract

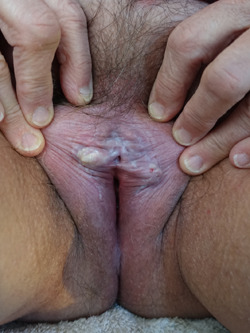

There is little information about the use of virtual consultation (VC) in patients with vulval disease. This prospective audit shows that VC is possible if the patient provides good quality imaging which is better if taken by a third party. It is most useful for those with established non‐cancer related diagnoses.

1

**What is already known about this topic?**

- There is no information about the use of virtual consultation (VC) in the field of vulval disease.

**What does this study add?**

- This is the first study evaluating VC in the field of vulval disease. It demonstrates that VC is possible in vulval conditions if patients are capable of providing high quality photographs for clinical assessment. However, it also suggests that follow‐up patients with established diagnoses may be most suitable for VC.

**What are the clinical implications of this work?**

- Although in‐person consultation will remain the standard of care, VC is feasible for vulval conditions. It is more appropriate for follow‐up patients with an established diagnosis. This may be particularly helpful for elderly patients with reduced mobility, those living in geographic areas with limited access to specialist vulval services, or in exceptional circumstances such as the COVID‐19 pandemic. Instructions for maximizing the quality of the patient submitted photos are warranted.

## 2 INTRODUCTION

Teledermatology (TD), the transfer of dermatological information through audio, visual and data communication, has been used for more than two decades, allowing patients living in remote areas or with reduced mobility to have access to dermatological care.^1^, ^2^, ^3^ TD has proved to be an acceptable substitute to in‐person consultations in terms of diagnostic efficacy, and reduction of waiting time to assessment, diagnosis and treatment,^4^, ^5^ providing a high degree of satisfaction to both patients and providers.^6^, ^7^, ^8^ TD is considered a valuable tool for the management of acne, atopic dermatitis, psoriasis, wounds, post‐operative follow‐up and to a lesser extent for diagnosis, treatment and follow‐up of skin cancers.^9^ However, there is almost no detail about how TD is applicable to patients with vulval disease. One study of 41 photos and 3 videos in 38 paediatric patients sent for consultation to a paediatric surgeon included one child with vulval synechiae.^10^ The telephone has been used as a communication tool in a long‐term post‐operative survey^11^ and for questionnaires in vulval pain,^12^ but rarely in a diagnostic capacity. One study showed that a self‐reported score for vaginal burning and vulval itching could be used to establish a diagnosis of vulvo‐vaginal candidiasis by telephone.^13^ However, the challenge of examining ‘sensitive areas of the body’ via TD in relation to hidradenitis suppurativa has been raised.^14^

During the early part of the COVID‐19 pandemic in 2020, most non‐emergency in‐person consultations were stopped and virtual consultations (VC), either by telephone or by video, were set up to protect both patients and physicians from viral exposure. Consequently, this led to an exponential rise in TD use^15^, ^16^ and offering it to patients who did not seem, at first, suitable candidates for this approach, such as patients with vulval conditions. Indeed, TD, specifically VC, poses several challenges when it comes to vulval conditions. VC does not allow an adequate examination of the vulva which is crucial for diagnosis; furthermore, vaginal, perianal, oral and general skin examination, which may be required to look for diagnostic clues at other sites, is not always possible. Photographs provided by the patient before or after the VC could compensate for the lack of a standard clinical examination. However, in contrast to other sites of the body, the vulva is not easily accessible for self‐photography. This may even be impossible for elderly patients with mobility or visual issues, or technical inability to use a smartphone. Furthermore, patients may be reluctant to send photos of their genitalia, even to a doctor. Asking for the help of a partner or relative is either embarrassing or impossible for patients living alone. Video recording of the vulva during a VC could also prove embarrassing for both the patient and the physician.

We report audit data from our experience of running specialist vulval services during the COVID‐19 pandemic using telephone or video consultations with and without supplementary imaging. Our objective was to assess the quality of VC in patients with vulval conditions, focussing on the amount and quality of supplementary imaging provided, the need for further consultation and physician satisfaction.

## 3 METHODS

This audit of VCs in vulval disease took place in two different settings—a specialist hospital‐based vulval clinic in London and two private office‐based specialist vulval clinics in Paris. All assessments were undertaken by a consultant dermatologist with a specialist interest in vulval disease. In London, in‐person outpatient consultations were suspended indefinitely at the end of March 2020, except for those with a suspected diagnosis of vulval cancer or emergency presentations. Patients with non‐acute presentations requiring review were instead offered telephone consultation, without any specific request to send phoographs, although this was discussed during the telephone consultation. In Paris, in‐person consultations were cancelled in the same way and patients were offered a video consultation (without live clinical examination) or alternatively, a telephone consultation with submission of clinical photographs of the vulva, via the Doctolib^®^ platform prior to the appointment. This is approved by both the French authorities and international standards of data protection and when patients register on Doctolib^®^, they must accept the Terms of Use and Privacy Policy in order to use the Doctolib^®^ services (Via Doctolib^®^, the patient data are stored in France [Paris] at an approved host which is certified by the French label ‘HDS’ [‘Hébergeur de Données de Santé’] in accordance with the law and the standards established by the ANS [‘Agence du Numérique en Santé’], in consultation with the CNIL [‘Commission nationale de l'informatique et des libertés’]. The hosting providers are also certified by the main international standards, including ISO/IEC 27001, and are audited annually by an independent body.).

Some patients proposed a vulval examination by video during the VC. This live video vulval examination was accepted by the physician if no photo was available or if the images were of poor quality.

During the 3 month period between March to May 2020, anonymized data on all consecutive VCs were collected prospectively using a standardized electronic database. Data collected included clinical information such as age and diagnosis, as well as information on the consultation, such as whether the consultation was a new or follow‐up appointment and whether supplementary imaging (photographs and/or videos) was submitted by the patient. Where relevant, the number of photographs sent by each patient was recorded and their collective quality was assessed according to a five‐point Likert‐type scale, rated from 1 (poor quality) to 5 (excellent quality). Data on outcomes such as physician certainty of the diagnosis in new patient consultations (‘certain’, ‘probable’ or ‘no diagnosis made’) and the need for further in‐person consultation were also recorded (‘immediate in‐person consultation required’, ‘no in‐person consultation required and discharged’ and ‘delayed follow‐up booked’). Global physician assessment of the perceived usefulness of VC was graded on a five‐point Likert‐type scale from 1 (‘useless’) to 5 (‘extremely useful’). Analyzed data were stratified according to the consultation type (new or follow‐up) and whether supplementary imaging (photographs or video) were received. Analyses were performed using SPSS^®^ and STATA 15.1 software and where data was missing for any given variable, these were excluded from the analysis.

## 4 RESULTS

A total of 316 patients from London (*n* = 199) and Paris (*n* = 117) were included. Mean age in all patients was 45.4 years (range 0.5–94 years) and was comparable when stratified according to clinical site (London mean age 47.4 years, standard deviation [SD] 21.1; Paris mean age 42.0, SD 15.0). Overall, 18.7% (*n* = 59) were new patient consultations and 81.3% (*n* = 257) were follow‐up. A higher proportion of consultations were for new patients in the Parisian clinics compared to London (45.3% vs. 3.0%).

**FIGURE 1:**
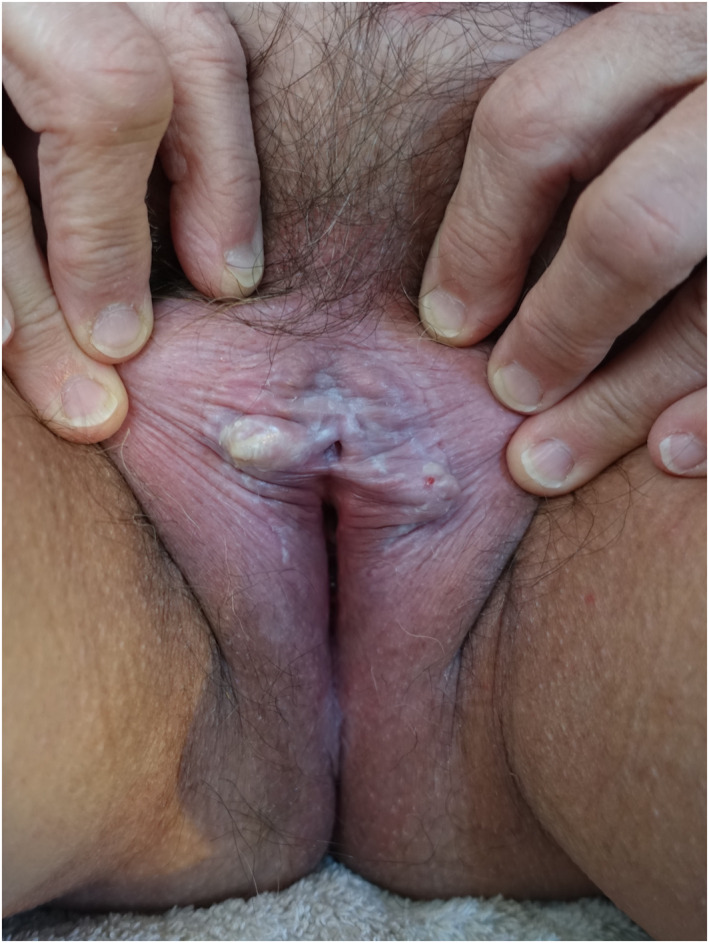
Hyperkeratotic lichen sclerosus—photograph taken by the husband. This patient required in‐person consultation for biopsy of the nodule.

**FIGURE 2:**
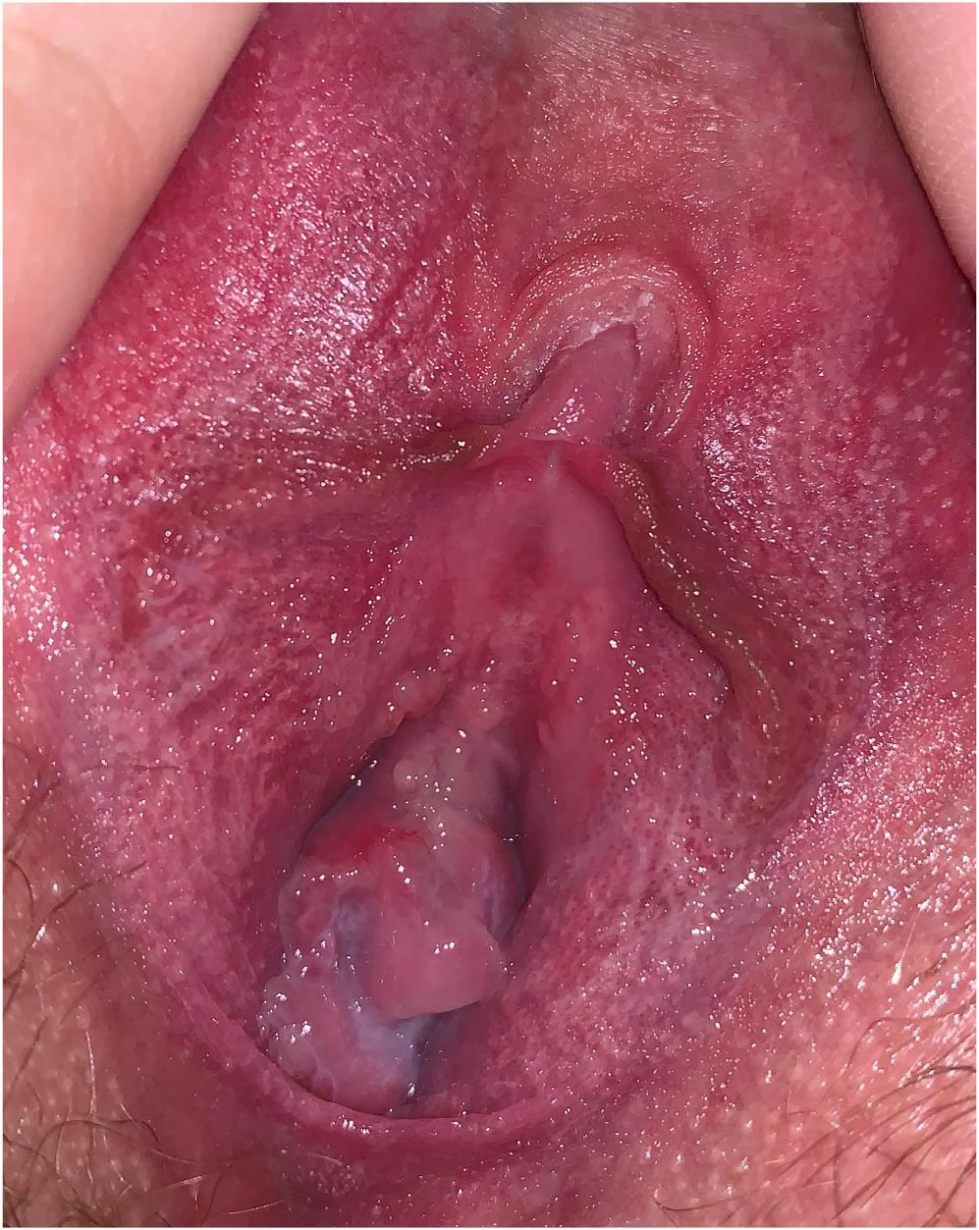
Normal variants—Fordyce spots and vestibular papillae—photograph taken by the patient. No further in‐patient consultation or treatment required.

Further data on the clinical and consultation characteristics are summarized in Table 1. Those who provided supplementary imaging as photographs and/or video recordings represented 28.5% (*n* = 90) of the total cohort. Notably, a higher proportion of patients attending for new consultations provided supplementary imaging (83.1%, *n* = 49) than those attending for follow‐up (16.0%, *n* = 41). Photographs alone were provided in 90.0% of cases, video alone in 3.3% or both video and photographs together in 6.7%. Amongst those who provided photographs only, 63.3% were taken by the patient themselves and 36.7% were taken by a third party, including a partner (27.9%), mother (3.8%), friend (1.3%), sister‐in‐law (1.3%) and carer (2.5%). The median number of photographs sent was 3 (range 1–20) with a median quality of 3 (interquartile range 1). The median quality of these photographs was significantly higher when they were taken by a third party (Figure 1) as opposed to the patient themselves (4 vs. 3, Mann‐Whitney *U*‐test, *p* < 0.0001). However, 16 of 49 patients (32.7%) who took photographs themselves provided images with a quality score ≥4 (Figure 2). Following discussion amongst the responsible clinicians, a consensus was reached that the quality of video recordings (*n* = 9) was generally poor.

**TABLE 1:** Clinical and consultation characteristics of patients seen for vulval conditions

	New		Follow‐up		Total		Missing
	Without imaging	With imaging	Without imaging	With imaging	Without imaging	With imaging	
Image type							
Photographs only	‐	45 (91.8)	‐	36 (87.8)	‐	81 (90.0)	0
Video and photographs	‐	3 (6.1)	‐	3 (7.3)	‐	6 (6.7)	0
Video only	‐	1 (2.0)	‐	2 (4.9)	‐	3 (3.3)	0
No. photographs per patient^a^	‐	3 (1–20)^b^	‐	3 (1–16)^b^		3 (1–20)^b^	0
Photograph quality^a^	‐	3 (1)^b^	‐	3 (1)^b^	‐	3 (1)^b^	1
Photographs taken by^a^							
Patient	‐	27 (61.4)	‐	23 (65.7)	‐	50 (63.3)	2
Partner	‐	10 (22.7)	‐	12 (34.3)	‐	22 (27.9)	2
Relative or friend	‐	5 (11.4)	‐	0 (0)	‐	5 (6.3)	2
Healthcare professional	‐	2 (4.6)	‐	0 (0)	‐	2 (2.5)	2
Diagnosis							
Lichen sclerosus	0 (0)	11 (22.5)	98 (45.4)	11 (26.8)	98 (43.4)	22 (24.4)	0
Vulvodynia	7 (70.0)	13 (26.5)	14 (6.5)	8 (19.5)	21 (9.3)	21 (23.3)	0
Eczema/lichen simplex	1 (10.0)	3 (6.1)	32 (14.8)	1 (2.4)	33 (14.6)	4 (4.4)	0
Lichen planus	0 (0)	1 (2.0)	22 (10.2)	4 (9.8)	22 (9.7)	5 (5.6)	0
Candidosis	1 (10.0)	0 (0)	7 (3.2)	5 (12.2)	8 (3.5)	5 (5.6)	0
Psoriasis	0 (0)	4 (8.2)	9 (4.2)	1 (2.4)	9 (4.0)	5 (5.6)	0
HSIL	0 (0)	0 (0)	10 (4.6)	2 (4.9)	10 (4.4)	2 (2.2)	0
Other/no diagnosis	1 (10.0)	17 (34.7)	24 (11.1)	9 (22.0)	25 (11.1)	26 (28.9)	0
Diagnostic certainty							
Sure	3 (30.0)	28 (57.1)	‐	‐	‐	‐	0
Probable	7 (70.0)	15 (30.6)	‐	‐	‐	‐	0
None	0 (0)	6 (12.2)	‐	‐	‐	‐	0
Outcome							
In‐person consultation	6 (60)	27 (55.1)	32 (14.8)	16 (39.0)	38 (16.8)	43 (47.8)	0
No in‐person consultation and discharged	4 (40)	22 (44.9)	54 (25.0)	24 (58.5)	58 (25.7)	46 (51.1)	0
Delayed follow‐up booked	0 (0)	0 (0)	130 (60.2)	1 (2.4)	130 (57.5)	1 (1.1)	0
Physician satisfaction	3 (1)^b^	4 (2)^b^	3 (1)^b^	5 (1)^b^	3 (1)^b^	4 (2)^b^	0

Abbreviation: HSIL, high‐grade squamous intraepithelial lesion.

^a^ Calculated for patients providing photographs alone.

^b^ Expressed as median (range).

Vulval conditions diagnosed and/or assessed during the consultations included lichen sclerosus (38.0%), vulvodynia (13.3%), eczema/lichen simplex (11.7%), lichen planus (8.5%), psoriasis (4.4%), candidiasis (4.1%) and high‐grade squamous intraepithelial lesions (3.8%). Amongst new patients, a higher diagnostic certainty (denoted as ‘certain’) was observed amongst those with supplementary imaging (photographs or video) compared to those without (57.1% vs. 30.0%), although a higher proportion of those with supplementary imaging also had ‘no diagnosis’ (12.2% vs. 0%) and Fisher's exact test demonstrated no association between the use of supplementary imaging and diagnostic certainty (*p* = 0.07). Despite their poor quality, the responsible clinicians reported that video consultation alone helped to establish a diagnoses of lichen sclerosus, candidiasis and psoriasis in three patients, whereas in six patients it helped to confirm diagnoses suspected on photos (vulvodynia—two cases, lichen simplex, warts, candidiasis, herpes simplex).

Twenty‐seven of 49 new patients (55.1%) with supplementary imaging required an immediate in‐person consultation to make a diagnosis and this was comparable to patients who did not provide imaging (60%). Amongst follow‐up consultations, a higher proportion of patients with supplementary imaging required immediate in‐person consultation (39.0% vs. 14.8%) or were discharged without in‐person consultation (58.5% vs. 25.0%) and only a few required deferral of their follow‐up appointment (2.4% vs. 60.2%). This observed association was statistically significant (*χ* ^2^ test, *p* < 0.001). Median physician satisfaction was higher in both new (4 vs. 3) and follow‐up (5 vs. 3) consultations when supplementary imaging was available and amongst follow‐up patients, this observed difference was statistically significant (Mann‐Whitney *U*‐test, *p* < 0.0001). Furthermore, median physician satisfaction amongst follow‐up patients varied significantly according to the working diagnosis (Table 2. Kruskall‐Wallis test, *p* = 0.0001), with median satisfaction scores ≥4 observed in candidiasis, lichen simplex/eczema and vulvodynia.

**TABLE 2:** Physician satisfaction amongst follow‐up patients, stratified according to diagnosis

	Physician satisfaction
Lichen sclerosus	3 (1)
Vulvodynia	4.5 (1)
Eczema/lichen simplex	4 (2)
Lichen planus	3 (1)
Candidosis	4 (1.5)
Psoriasis	3.5 (2)
HSIL	2 (1)
Other/no diagnosis	3 (2)

*Note*: Expressed as median (interquartile range).

Abbreviation: HSIL, high‐grade squamous intraepithelial lesion.

## 5 DISCUSSION

The age range of this patient cohort (0.5–94 years) indicates that VC is a possible alternative to in‐person consultations for vulval conditions amongst patients of all ages. Although only 28.5% of the cohort provided supplementary imaging (photograph or video) as part of their VC, this was likely attributable to the fact that patients from the London clinic (63.0% of the total cohort) were not specifically invited to submit photographs at the time when the VC was offered. However, when patients were invited to send photographs, as in the Parisian clinics, the rate of available photographs made available increased to 68.4%. Specific invitations to submit photos before VC for vulval conditions could, therefore, improve the effectiveness of the consultation. Nevertheless, technical limitations (poor Internet resources, difficulty with self‐photography, absence of a third party to help), as well as cultural, religious and psychological barriers to sending personal images of the genitalia may exist that will ultimately influence the rate of image provision. Furthermore, safe and user‐friendly mechanisms by which sensitive images of the genitalia can be transferred to clinicians must be put in place if VC were to be offered regularly. This would include advice for patients about encryption and consent for storage of images if they used an alternative method from the secure recommended platform.

This study also demonstrated that it is possible for patients to provide high‐quality photographs for vulval VC, particularly if these photographs are taken by a third party. Twenty‐nine (36.7%) patients were helped a by a third party and provided photos which were of higher quality than those taken by the patient themselves. However, despite the technical difficulty of vulval self‐photography, 32.7% of patients were able to provide the physician with high quality pictures (≥4 on a five‐point Likert‐type scale). Consequently, precise written instructions about how to take vulval pictures could help patients, relatives or carers, to improve the quality of the images provided and increase the success of vulval VC in the future. Although not invited to do so by the physician, nine patients offered to be examined by video. Despite the poor quality of video, this tool helped to assess the diagnosis of various conditions either alone (lichen sclerosus, candidiasis, psoriasis) or in conjunction with photos (lichen simplex, vulvodynia, herpes, warts, candidiasis).

Amongst new patient consultations, supplementary imaging did not improve diagnostic certainty or reduce the need for in‐person consultations to confirm the suspected diagnoses. These observations suggest that there remain challenges in assessing new patients via vulval VC and that physicians may not be comfortable in making a definitive diagnosis in the absence of an in‐person examination. Although not assessed in this audit, patient perception and satisfaction around the clinical assessment might also be lower in this context. In follow‐up consultations, however, supplementary imaging led to more definitive clinical decision‐making regarding the need for follow‐up, with more patients being either seen in‐person or discharged from services with advice and fewer deferred follow‐up appointments. This is probably due to the fact that the diagnosis was known and so it was easier to make a decision or defer the follow‐up consultation knowing the diagnosis and what the potential cause for further symptoms may be. Likewise, physician satisfaction was significantly higher amongst follow‐up patients who provided supplementary imaging, in particular for candidiasis, lichen simplex/eczema or vulvodynia. These observations suggest, therefore, that patients with established, cancer‐unrelated vulval diagnoses are likely the best candidates for VC.

## 6 STRENGTHS AND LIMITATIONS

This is to our knowledge, the first description of VC for vulval conditions. The study included a large cohort of patients in two different settings and involved three specialists in vulval conditions. There are limitations in that the physicians involved in this study were all experienced in the field of vulval disease. Therefore, our results may not be applicable to those who have less experience in the assessment and management of women with vulval symptoms. The numbers of patients who provided supplementary imaging or attended as new consultations were also small and this may have limited statistical power when analyses were undertaken. In addition, the degree of physician satisfaction and assessment of quality of the photographs would have benefitted from more precise definitions to reduce inter‐observer variation. Further studies should include patient satisfaction which was not formally measured in this study. As for TD, accurate and measurable indicators should be defined including processes, quality, cost and both physician and patient satisfaction.^17^, ^18^ The first aim of this pilot study was to establish feasibility of VC for vulval conditions, with and without imaging. The efficiency was measured only through the clinician diagnostic certainty and the need for further in‐person consultation. We did not evaluate the diagnostic accuracy by comparison with in‐person consultations as the number of patients needing in‐person consultation after photograph use was small and would therefore not reach statistical significance in this cohort.

## 7 CONCLUSION

Although not obvious when first considered, VC is possible for patients with vulval disease. To improve the quality of the patient submitted images, precise technical instructions should be provided. As the vulva is not easily accessible for self‐photography, the help of a third party is preferable, although not always feasible. Follow‐up patients with an established cancer‐unrelated diagnosis are most suitable for VC. Further studies are needed to evaluate diagnostic accuracy.

## CONFLICT OF INTEREST

The authors declare that there is no conflict of interest that could be perceived as prejudicing the impartiality of the research reported.

## AUTHOR CONTRIBUTIONS

**Fiona M. Lewis**: Conceptualization (equal); Data curation (equal); Formal analysis (equal); Methodology (equal); Writing – original draft (equal); Writing – review & editing (equal). **Sheila M. McSweeney**: Formal analysis (equal); Writing – original draft (equal); Writing – review & editing (equal). **Jeanne Wendling**: Data curation (equal); Writing – original draft (equal); Writing – review & editing (equal). **Micheline Moyal‐Barracco**: Conceptualization (equal); Data curation (equal); Formal analysis (equal); Methodology (equal); Writing – original draft (equal); Writing – review & editing (equal).

## ETHICS STATEMENT

Not applicable.

## Data Availability

The data that support the findings of this study are available from the corresponding author upon reasonable request.
